# Frequency of Revaccination against Smallpox

**DOI:** 10.3201/eid0911.020820

**Published:** 2003-11

**Authors:** Samuel Baron, Jingzhi Pan, Joyce Poast

**Affiliations:** * University of Texas Medical Branch, Galveston, Texas, USA

Recent suggestions to revise guidelines that recommend extending the time for revaccination beyond 10 years may be based on insufficient and conflicting evidence of persistence of immunity (*1*,*2*). The evidence that cell-mediated immunity and neutralizing antibody persist after one vaccination is conflicting (*2*–*5*). This residual immunity is often low, and its protective activity in vivo is unclear. Similarly, in vivo reports of durable immunity to smallpox (*1*) were not sufficiently controlled, and short persistence of resistance to dermal challenge with the antigenically related vaccinia virus has been reported (*3*,*6*).

We confirmed the previous report (*5*) that the residual antivaccinia virus titers of serum samples from singly vaccinated adults are low (average 32) (Table). The titer of normal commercial immunoglobulin (Ig) (Panglobulin) (11 times concentrated sera) was 150 U/mL, which when calculated to include the 11-fold concentration, confirms the low residual titers.

**Table:** Residual vaccinia virus neutralizing titers of serum from vaccinated and unvaccinated persons

Participant no.	Y after vaccination	Neutralizing titer
Single-vaccinated persons		
A	47	10
B	50	26
C	45	27
D	40	34
E	40	43
Ordinary commercial immunoglobulin (Panglobulin)		150
Unvaccinated persons		
H6	—^a^	<10
H9	—	<10
C2	—	<10
C4	—	<10
H1	—	10
H2	—	10
H13	—	10
C1	—	10
C3	—	10
H7	—	10
HL	—	20
H3	—	20
H4	—	20
H5	—	20
H10	—	20
H11	—	20
H12	—	20
C7	—	20
H8	—	30

^a^—, Not applicable.

The titers of the control, unvaccinated persons, averaged 14, raising questions about the importance and specificity of the residual antibody in vaccinated persons. We determined that the persistent neutralizing activity is mainly IgG antibody in serum from both single-vaccinated persons and ordinary commercial IgG, since sequential absorption with protein G beads and anti-IgG beads reduced the titers 80%. However, the neutralizing activity in unvaccinated control serum may not be mainly IgG antibody since neutralizing activity was reduced by an average of 48%, favoring nonspecific inhibitors. Studies of these nonspecific inhibitors and possible cross-immunizing antigens in the environment should be conducted to explain the occurrence of neutralizing activity in serum of unvaccinated persons.

To determine whether the low residual titers in sera from single vaccinated persons protected in vivo against a systemic infection, mice were pretreated subcutaneously with 1 mL of either 1) serum from a single-vaccinated study participant containing the low 10 U/mL neutralizing activity (patient A), 2) serum from a single-vaccinated person containing the higher 43 U/mL (patient E), or 3) normal commercial Ig containing 150 U/mL and challenged 24 hours later with one LD_100_ vaccinia virus, strain IHD-E, intraperitoneally. The 1 mL of serum injected into the mice is estimated to provide its original titer in the mouse. The lowest titer serum (10 U/mL) did not protect the mice against lethal systemic infection, whereas the highest titer serum (43 U/mL) and the commercial Ig (150 U/mL) protected 50% of the mice. Thus, the levels of residual antibody in vaccinated persons are either not protective or only partially protective in mice. Consistent with the reported protection by the higher levels of antibody, vaccinia immune globulin (VIG), which contains 500 neutralizing U/mL, is effective under some conditions (*7*–*10*). As a positive control for protection in this animal model, 100 μg of the interferon inducer Poly I:CLC protected 100% of the mice. Undetermined and requiring study is whether active immunity might be protective through an anamnestic response. The animal models of poxvirus infection have been used to evaluate immunity, but no generally established laboratory surrogate exists for immunity to smallpox virus itself. Persistence of effective humoral immunity after a single vaccination and its ability to effectively protect in vivo remain questionable.
